# STAT2-directed pathogen responses

**DOI:** 10.18632/oncotarget.5266

**Published:** 2015-08-26

**Authors:** Hans Bluyssen

**Affiliations:** Department of Human Molecular Genetics, Institute of Molecular Biology and Biotechnology, Faculty of Biology, Adam Mickiewicz University, Poznan, Poland

**Keywords:** Immunology and Microbiology Section, Immune response, Immunity, interferon response, STAT2 signaling, host-pathogen interaction

**Figure 1:**
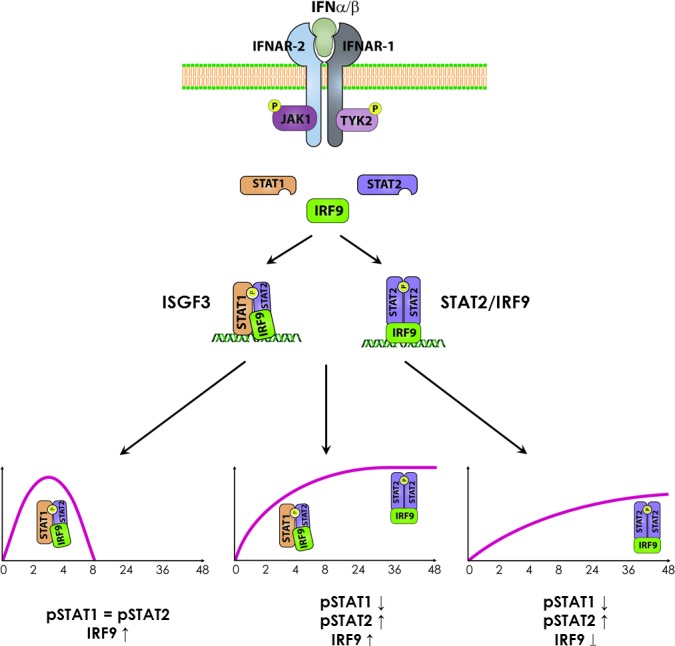
Abundance and phosphorylation kinetics of ISGF3 components dictate the nature and duration of IFNa responses Type I IFNs induce gene expression in an ISGF3-dependent manner, or alternatively, through STAT2/IRF9. In cell types with a transient STAT1 and STAT2 phosphorylation pattern and elevated IRF9 expression, ISGF3 is the pre-dominant mediator of IFNα signalling. In contrast, in cells with elevated levels of STAT2 and IRF9, prolonged STAT2 phosphorylation and transient activity of STAT1, ISGF3 (first) and STAT2/IRF9 (later) could facilitate a robust and prolonged IFN response. Finally, under circumstances of elevated levels of STAT2 and low basal levels of IRF9, prolonged STAT2 phosphorylation and transient activity of STAT1, STAT2/IRF9 alone could mediate a prolonged IFN response

Interferons (IFNs) are a subset of cytokines that mediate innate immune responses and provide a robust first line of defense against invading pathogens. IFNs represent a family of molecules which can be divided into three main sub-families: Type I, Type II and Type III [1]. Type I IFNs predominantly consist of IFN*α* and IFN*β* subtypes, Type II consists of the single IFN*γ* type, while Type III comprises IFN*λ*1, IFN*λ*2 and IFN*λ*3 [3]. IFN molecules bind to cell surface receptors and initiate a signaling cascade through the Janus kinase signal transducer and activator of transcription (JAK-STAT) pathway, resulting in the transcriptional regulation of hundreds of IFN-stimulated genes (ISGs) [2]. STAT1 and STAT2 are key mediators of responses to Type I and Type III IFN. Together with the DNA-binding protein interferon regulatory factor (IRF) 9 they form a heterotrimeric transcription complex termed interferon-stimulated gene factor 3 (ISGF3) that binds to the interferon-stimulated response element (ISRE) in ISG promoters (Figure 1). STAT1 homodimers facilitate transcriptional responses to all types of IFN by directly activating genes containing the IFN*γ* -activated site (GAS) DNA element [1,2]. Collectively, this leads to a powerful antiviral state that is capable of controlling infections of positive-, negative-, and double-stranded RNA viruses and DNA viruses, as well as intracellular bacteria and parasites. However, viruses can still replicate and cause disease *in vivo*, because they have some strategy for at least partially circumventing the IFN response. For example, evidence exists that certain viruses have the capacity to block the activation of specific components of the IFN-induced signaling cascade and thus the presence of alternative antiviral responses could provide the host with distinct survival advantages [3].

Indeed, evidence is accumulating that the role of STAT2 and IRF9 in the regulation of specific transcriptional programs is not restricted to their involvement in the classical ISGF3 complex [4]. Previously, we showed that STAT2 is also capable of forming homodimers when phosphorylated in response to IFN*α* [5]. These STAT2 homodimers were shown to interact with IRF9 and form the ISGF3-like complex STAT2/IRF9 that activates transcription of ISRE-containing genes in response to IFN*α* [5] (Figure 1). Different *in vitro* and *in vivo* studies have subsequently pointed to the existence of a STAT1-independent IFN*α* signalling pathway, where STAT2/IRF9 can potentially substitute for the role of ISGF3 [4].

Recently, we provided further insight into the genome-wide transcriptional regulation and the biological implications of STAT2/IRF9-dependent IFN*α* signalling as compared to ISGF3 [6]. In STAT1-defeicient human and mouse cells stably overexpressing STAT2 we observed that the IFN*α*-induced expression of typical ISGs correlated with the kinetics of STAT2 phosphorylation, and the presence of a STAT2/IRF9 complex. Subsequently, we identified ∼120 known antiviral ISRE-containing ISGs commonly up-regulated by STAT2/IRF9 and ISGF3. The STAT2/IRF9-directed ISG expression profile was prolonged as compared with the early and transient response mediated by ISGF3. Finally, STAT2/IRF9 was able to trigger an antiviral response upon encephalo myocarditis virus and vesicular stomatitis Indiana virus. We believe that the lower DNA-affinity of the STAT2/IRF9 complex as compared with ISGF3 [5], requires abundance of STAT2 and IRF9 protein and correlates with the delayed and prolonged nature of its IFN*α*-mediated activity. Therefore, in the absence of STAT1 a certain threshold amount of STAT2 and IRF9 must be reached to allow STAT2 phosphorylation and STAT2/IRF9-mediated transcription.

A parallel study performed by Abdul-Sater et al. [7] demonstrated that, in primary Stat1−/− BMM cells, STAT2 can associate with IRF9 to drive the delayed expression of a subset of ISGs important in the innate response to L. pneumophila, Dengue virus, as well as potentially other viruses. In this study, STAT2 was identified as a key component of the STAT1-independent mechanism of protection against DENV infection in mice, and demonstrated that both STAT1 and STAT2 possess the ability to independently limit the severity of DENV pathogenesis. For many viruses, inhibition of STAT-mediated signaling is a major mechanism to evade antiviral responses. Therefore, these data suggest that DENV-mediated inactivation of STAT1 function alone is not sufficient to neutralize antiviral responses, and it is tempting to speculate that the STAT2/IRF9 pathway evolved as a backup response to defend against pathogens that impede STAT1 activity (e.g., Paramyxovirus) [3]. It also emphasizes the importance of DENV mechanisms (and that of other viruses) to specifically target host STAT2 function.

These data coincide with the hypothesis that abundance and phosphorylation kinetics of ISGF3 components dictate the nature and duration of IFNα responses (Figure 1). In cell types with a transient STAT1 and STAT2 phosphorylation pattern ISGF3 is the pre-dominant mediator of IFN*α* signalling. In contrast, in cells with elevated levels of STAT2 and prolonged STAT2 phosphorylation STAT2/IRF9 can either coexist with the classical ISGF3 complex or act solely, depending on the level of STAT1 and IRF9 (Figure 1). These situations are very likely to be cell-type-specific, and could provide a level of redundancy to certain cells to ensure effective induction of an antiviral state.

The physiologic situations in which IFN-dependent gene regulation occurs in the absence of STAT1 remain undefined. This situation will occur if STAT1 is depleted by chronic stimulation, during severe immune stress, or inhibited during viral infection, providing an additional setting in which the STAT2/IRF9 pathway could offer a backup response. Further elucidating the mechanisms and physiologic consequences of the alternative STAT2/IRF9 IFN signalling pathway, as compared with ISGF3, in different cell types and how different viruses cope with it is of fundamental interest, but may also have practical application in the design and manufacture of attenuated virus vaccines and the development of novel antiviral drugs.
